# Sustained Drug Treatment Alters the Gut Microbiota in Rheumatoid Arthritis

**DOI:** 10.3389/fimmu.2021.704089

**Published:** 2021-10-14

**Authors:** Liyan Mei, Zhihua Yang, Xiaolin Zhang, Zehao Liu, Maojie Wang, Xiaodong Wu, Xiumin Chen, Qingchun Huang, Runyue Huang

**Affiliations:** ^1^ The Second Affiliated Hospital of Guangzhou University of Chinese Medicine (Guangdong Provincial Hospital of Chinese Medicine), Guangzhou, China; ^2^ Center for Molecular Medicine, University Medical Center Utrecht, Utrecht, Netherlands; ^3^ State Key Laboratory of Dampness Syndrome of Chinese Medicine (The Second Affiliated Hospital of Guangzhou University of Chinese Medicine), Guangzhou, China; ^4^ Guangdong Provincial Key Laboratory of Clinical Research on Traditional Chinese Medicine Syndrome, Guangzhou, China; ^5^ Guangdong-Hong Kong-Macau Joint Lab on Chinese Medicine and Immune Disease Research, Guangzhou University of Chinese Medicine, Guangzhou, China

**Keywords:** rheumatoid arthritis, gut microbiota, drug treatment, methotrexate, traditional Chinese medicine, leflunomide

## Abstract

Several studies have investigated the causative role of the microbiome in the development of rheumatoid arthritis (RA), but changes in the gut microbiome in RA patients during drug treatment have been less well studied. Here, we tracked the longitudinal changes in gut bacteria in 22 RA patients who were randomized into two groups and treated with Huayu-Qiangshen-Tongbi formula (HQT) plus methotrexate (MTX) or leflunomide (LEF) plus MTX. There were differences in the gut microbiome between untreated (at baseline) RA patients and healthy controls, with 37 species being more abundant in the RA patients and 21 species (including *Clostridium celatum*) being less abundant. Regarding the functional analysis, vitamin K2 biosynthesis was associated with RA-enriched bacteria. Additionally, in RA patients, alterations in gut microbial species appeared to be associated with RA-related clinical indicators through changing various gut microbiome functional pathways. The clinical efficacy of the two treatments was further observed to be similar, but the response trends of RA-related clinical indices in the two treatment groups differed. For example, HQT treatment affected the erythrocyte sedimentation rate (ESR), while LEF treatment affected the C-reactive protein (CRP) level. Further, 11 species and 9 metabolic pathways significantly changed over time in the HQT group (including *C. celatum*, which increased), while only 4 species and 2 metabolic pathways significantly changed over time in the LEF group. In summary, we studied the alterations in the gut microbiome of RA patients being treated with HQT or LEF. The results provide useful information on the role of the gut microbiota in the pathogenesis of RA, and they also provide potentially effective directions for developing new RA treatments.

## Introduction

Rheumatoid arthritis (RA) is a common chronic systemic inflammatory autoimmune disease characterized by the production of autoantibodies that target various molecules (1). Painful joint swelling and morning stiffness (2) are typical clinical manifestations of RA, which severely impair physical function and quality of life. The pathogenesis of RA is complex and involves several risk factors, including susceptibility genes, gender, and environmental factors. Increasingly, studies are revealing that microbiota alterations (3–5), particularly changes in the gut and oral microbiomes, are important environmental risk factors in RA development (6).

Driven by advances in shotgun sequencing technology, the burgeoning field of metagenomics has begun to uncover the impact of microbes on RA. *Haemophilus* spp. was decreased and *Lactobacillus salivarius* was increased in RA patients compared to healthy controls (HCs) in the gut, saliva, or dental microbial communities, but microbiome dysbiosis was partially resolved by RA DMARDs treatment (7). A recent study reported a decreased abundance of *Enterobacter*, *Odoribacter*, *Lactobacillus*, and *Alloprevotella* and an increased abundance of the genera *Bacteroides* and *Escherichia-Shigella* in a RA cohort in China (8), while there was an increased abundance of the genus *Prevotella* (e.g., *Prevotella denticola*) in Japanese RA patients. *Blautia*, *Akkermansia*, and *Clostridiales* were increased in anti-citrullinated peptide antibody (ACPA)-positive RA patients compared to ACPA-negative RA patients (9).

Additionally, several studies have indicated that periodontitis is closely associated with RA (10). Specific oral bacteria that are causative agents of periodontal disease have been shown to significantly influence the progression of RA. Members of the oral microbiome, such as *P. gingivalis*, *Prevotella intermedia*, and *Aggregatibacter actinomycetemcomitans*, play roles in the onset of RA *via* mechanisms such as direct or indirect modulation of citrullination and altering T cell-mediated adaptive immunity.

Increasing experimental and clinical evidence also suggests that the gut microbiome composition and function are affected by RA treatment. For example, a study by Picchianti-Diamanti et al. (11) showed that the tumor necrosis factor alpha (TNF-α) inhibitor etanercept can alter microbial communities and at least partially improve the microbiota in RA patients. Similarly, in mice with collagen-induced arthritis, etanercept reduced the abundance of *Escherichia/Shigella* and increased the abundance of *Lactobacillus, Clostridium XIVa*, and *Tannerella* (12). Additionally, antibiotic-induced partial depletion of the gut microbiota aggravated arthritis symptoms in a RA mouse model (13). Regarding natural compounds, *Clematis* triterpenoid saponins can alleviate arthritis-associated gut microbial dysbiosis and thereby improve arthritic disease indices (14). Recently, the emergence of data on microbiomes associated with different treatments has provided new insights regarding the associations among RA treatments, the gut microbiota, and clinical outcomes.

Huayu-Qiangshen-Tongbi formula (HQT) is a traditional Chinese medicine (TCM) formula adapted from classical Chinese medicine that has been widely used in clinical practice for the treatment of RA. In particular, the clinical application of HQT in combination with DMARDs is highly effective in the treatment of RA. Interestingly, we have also confirmed the effectiveness and possible mechanism of HQT in the treatment of RA through clinical trials and experimental studies. In the early stages, we implemented a retrospective clinical study and an investigator-initiated randomized clinical study with methotrexate (MTX) as the baseline drug and leflunomide (LEF) as the control, both of which suggested that “HQT+MTX” may have similar or even better clinical efficacy and tolerability than “LEF+MTX”. Importantly, the “HQT+MTX” group had fewer adverse events compared to the “HQT+LEF” group (15, 16). Along with the successful clinical trial of HQT, we have also conducted in-depth research on the mechanism of HQT for the treatment of RA and found that the mechanisms underlying the therapeutic effects of HQT on RA are closely related to its modulation of lncRNA uc.477 and miR-19b (17). In addition, pharmacological studies have also confirmed that HQT can treat RA by anti-inflammatory and regulating the body’s bone metabolism (18). In this study, the herbal composition and dosage of HQT were detailed in a previous study (16, 18).

Recent studies have sought to determine the gut microbiome factors that promote RA development. However, few studies have tracked the gut microbiota alterations in RA patients during treatment. In this study, we tracked the gut microbiota alterations in RA patients being treated with either HQT formula plus MTX or LEF plus MTX over 6 months. We aimed to assess the effects of the different treatments on the gut microbiome in RA patients.

## Materials and Methods

### Participant Recruitment and Ethics Statement

RA patients and healthy donors (44 participants) were recruited from the Second Affiliated Hospital of Guangzhou University of Chinese Medicine (Guangdong Provincial Hospital of Chinese Medicine) between August 2016 and September 2018. These included 22 RA patients who fulfilled the 2010 revised criteria of the American College of Rheumatology (ACR) for RA (19) and 22 ethnicity-, sex-, and age-matched individuals with no personal or family history of rheumatic diseases, who served as healthy controls (HCs) (**Table S1**).

After baseline sample collection, the 22 RA patients were randomized into two groups; 13 RA patients received the traditional Chinese medicine Huayu-Qiangshen-Tongbi (HQT) decoction (once every 2 days), while the other 9 RA patients received oral leflunomide (LEF; 20 mg/day). All patients received oral methotrexate (MTX; 10–15 mg/week). Each patient was continuously treated for 6 months. Fecal and blood samples were collected at baseline and in the first (M1), third (M3), and sixth (M6) months after treatment, along with detecting other RA-related clinical indices.

This study was reviewed and approved by the ethics committee of Guangdong Provincial Hospital of Chinese Medicine (no. B2016-076-01) and registered with the World Health Organization clinical trial registry (no. ChiCTR-INR-16009031). All participants provided their written informed consent to participate in this study before baseline sample collection. The 22 RA patients were part of a clinical study published in September 2020 (16).

### Metagenome Sequencing and Analysis

A total of 110 fecal samples from the 44 participants underwent metagenomic sequencing. Total bacterial DNA was extracted from the fecal samples using a NucleoSpin^®^ Soil kit (Macherey-Nagel, Düren, Germany) following the manufacturer’s instructions. A HiSeq X Ten sequencer (Illumina, San Diego, CA, USA) yielded 1,036.69 Gb of paired-end reads. After quality control and removal of host (human) reads, a mean ± SD of 8.18 ± 0.82 Gb of reads/sample remained for bioinformatics analysis. Taxonomic and functional profiling of the metagenomes were performed using MetaPhlAn2 (v2.0) and HUMAnN2 (v0.11.2), respectively. Detailed methods and parameters are provided in the **Supplementary Material**.

The origin of each species was determined using Integrated Microbial Genomes (IMG) data (http://img.jgi.doe.gov/cgi-bin/w/main.cgi). First, the bacterial species “Host Name” had to be “Homo sapiens”. Second, if the “Isolation” field contained oral components, e.g., “saliva”, “dental plaque”, or “nasopharynx”, the species was considered to be of oral origin. If it contained intestinal components, e.g., “feces” or “gastrointestinal tract”, the species was considered to be of fecal origin. In other cases, the species was considered to be of other or unknown origin.

### Statistical Analysis

The alpha diversity of the groups was estimated at the gene family and species level. Beta diversity between groups was estimated based on Bray–Curtis distance at the gene family level using the vegdist function in the vegan R package. Permutational multivariate analysis of variance (PERMANOVA) was also performed on the gene family profiles using the adonis function in the vegan R package, and the permuted P value was based on 9,999 permutations. Significantly affected taxa and MetaCyc pathways between the groups were identified by Wilcoxon rank-sum test, based on P < 0.05. Significantly affected Kyoto Encyclopedia of Genes and Genomes (KEGG) pathways were identified using the reporter score (20, 21) based on |reporter score| > 1.65. The Jonckheere-Terpstra test was used to investigate the trends in RA-associated bacterial species over time. The correlations among the relative abundances of species, gut microbiome KEGG functional pathways, and RA-related clinical indices were calculated by Spearman’s rank correlation analysis and visualized using the ComplexHeatmap R package. Cross-validated random forest modeling (using randomForest 4.6-14 R package) was performed based on the relative abundances of species in the samples (21). In 5 trials of 10 fold cross-validated model, all bacterial species were sorted according to the importance of their variable and added to the model in turn. Then the cross-validation error curves were averaged and the minimum error in the averaged curve plus the s.d. at the point was used as the cut-off for feature selection. All bacterial species set with an error less than the cut-off were listed and the set with the smallest number of bacterial species was selected as the optimal set. Enterotype analysis of samples was performed based on the genus relative abundance profile as described by Arumugam et al. (22). Jensen–Shannon divergence (JSD) distance metric of samples was calculated, and clustered using the PAM clustering algorithm. The optimal number of clusters was determine by Calinski–Harabaze (CH). Principal component analysis (PCA) was preformed to visualize samples distances using ‘ade4’ package in R. All statistical analyses were carried out in R (v3.5.0).

## Results

### General Characteristics of Study Cohorts

The study cohorts comprised 22 HCs and 22 RA patients, and their baseline characteristics are shown in **Table 1**. No significant differences in age, gender, or body mass index (BMI) were observed between the HC and RA groups. As expected, the RA diagnostic markers rheumatoid factor (RF) and anti-cyclic citrullinated peptide (anti-CCP) were significantly higher in RA patients than HCs. The inflammatory markers C-reactive protein (CRP) and erythrocyte sedimentation rate (ESR) were also significantly higher in RA patients than HCs. In contrast, no significant differences were observed in the other blood parameters except alanine transaminase (AST), which was lower in RA patients though still in the normal range in both groups.

**Table 1:** Baseline characteristics of healthy controls (HC; n = 22) and rheumatoid arthritis (RA) patients (n = 22).

Clinical factor	HCs; n = 22	RA patients; n = 22	Adjusted P value
Age, year^*^	48.5 ± 13.3	48.2 ± 11.5	1.000
Gender, female^§^	19 (86.4%)	19 (86.4%)	1.000
Height, cm^*^	158.7 ± 6.2	160.4 ± 5.4	0.327
Weight, kg^*^	52.2 ± 6.8	55.8 ± 6.4	0.203
BMI, kg/m^2*^	20.7 ± 1.8	21.7 ± 2.3	0.224
WBC count, 10^9^/L^*^	6.2 ± 1.3	7.4 ± 2.6	0.203
HB, g/L^*^	124.8 ± 17.2	127.6 ± 39.3	0.554
BUN, mmol/L^*^	4.8 ± 1.4	4.6 ± 1.4	0.687
Cr, μmol/L^*^	66.1 ± 14.7	62.9 ± 10.7	0.687
PLT count, 10^9^/L^*^	266.9 ± 61.8	303.5 ± 103.6	0.229
ALT, U/L^*^	18.3 ± 18.3	14.4 ± 11.0	0.283
AST, U/L^*^	20.7 ± 7.1	15.2 ± 2.9	0.002
hs-CRP, mmol/L^*^	1.2 ± 1.6	23.8 ± 20.3	<0.001
ESR, mm/h^*^	22.4 ± 14.8	59.5 ± 27.2	<0.001
RF, IU/mL^*^	7.5 ± 2.8	201.6 ± 269.1	<0.001
Anti-CCP, U/mL^*^	0.6 ± 0.2	102.1 ± 80.2	<0.001

*mean ± SD, ^§^n (%).

BMI, body mass index; WBC, white blood cell; HB, hemoglobin; BUN, blood urea nitrogen; Cr, creatinine; PLT, platelet; ALT, alanine transaminase; AST, aspartate transaminase; hs-CRP, high-sensitivity C-reactive protein; ESR, erythrocyte sedimentation rate; RF, rheumatoid factor; anti-CCP, anti-cyclic citrullinated peptides.

The baseline characteristics of the 13 MTX+HQT-treated and 9 MTX+LEF-treated RA patients in the study cohort are shown in **Table 2**. There were no significant differences in age, gender, height, and BMI between the HQT and LEF groups of RA patients, except for weight (*P*=0.044). Undoubtedly, the differences in RA-specific diagnostic markers, inflammatory markers, and other blood markers (RF, Anti-CCP, hs-CRP, ESR, WBC, HB, BUN, Cr, PLT, ALT, AST) between the two treatment groups were also not statistically significant.

**Table 2:** Baseline characteristics of MTX+HQT-treated (n = 13) and MTX+LEF-treated (n = 9) RA patients.

Clinical factor	MTX+HQT; n = 13	MTX+LEF; n = 9	Adjusted P value
Age, year^*^	50.4 ± 10.4	45.1 ± 12.8	0.300
Gender, female^§^	11 (84.6%)	8 (88.9%)	0.787
Height, cm^*^	161.2 ± 6.6	159.2 ± 3.1	0.416
Weight, kg^*^	58.1 ± 4.4	52.6 ± 7.6	0.044
BMI, kg/m^2*^	22.4± 2.0	20.7 ± 2.5	0.087
WBC count, 10^9^/L^*^	7.4 ± 2.8	7.4 ± 2.4	0.988
HB, g/L^*^	133.1 ± 50.0	119.7 ± 13.5	0.444
BUN, mmol/L^*^	4.8 ± 1.4	4.3 ± 1.5	0.370
Cr, μmol/L^*^	64.0 ± 11.2	61.3 ± 10.3	0.578
PLT count, 10^9^/L^*^	296.2 ± 122.0	314.2 ± 75.2	0.698
ALT, U/L^*^	14.9 ± 13.4	13.7± 6.7	0.799
AST, U/L^*^	15.0 ± 2.6	15.6 ± 3.5	0.674
hs-CRP, mmol/L^*^	22.5 ± 17.7	25.7 ± 24.7	0.724
ESR, mm/h^*^	67.9 ± 21.9	47.4 ± 30.7	0.082
RF, IU/mL^*^	222.4 ± 302.0	171.6 ± 227.1	0.674
Anti-CCP, U/mL^*^	97.0± 87.8	108.9 ± 73.4	0.746

*mean ± SD, ^§^n (%).

BMI, body mass index; WBC, white blood cell; HB, hemoglobin; BUN, blood urea nitrogen; Cr, creatinine; PLT, platelet; ALT, alanine transaminase; AST, aspartate transaminase; hs-CRP, high-sensitivity C-reactive protein; ESR, erythrocyte sedimentation rate; RF, rheumatoid factor; anti-CCP, anti-cyclic citrullinated peptides.

### Alterations in Gut Microbes of RA Patients

Based on the Shannon index, there was no significant difference in alpha diversity between the RA and HC groups. There was no significant difference in the number of species (**Figure S1A** and **Table S8**). There were also no significant differences in microbial community composition based on principal coordinate analysis (PCoA) and PERMANOVA at the gene family level (**Figure S1B** and **Table S9**). Furthermore, for the separate clusters of PCoA1 in **Figure S1B**, we found by individual gut type analysis that the main reason was the different gut types among individuals.Two clusters, one with *g_Prevotella* as the dominant species (HC=3; RA=6) and the other with *g_Bacteroides* as the dominant species (HC=19; RA=16). The results of Fisher’s exact test showed p=0.4566 for different gut types in HC and RA individuals, indicating that there was no significant difference in the frequency of different gut types in the two groups (**Figure S1C**).

**Figure 1:**
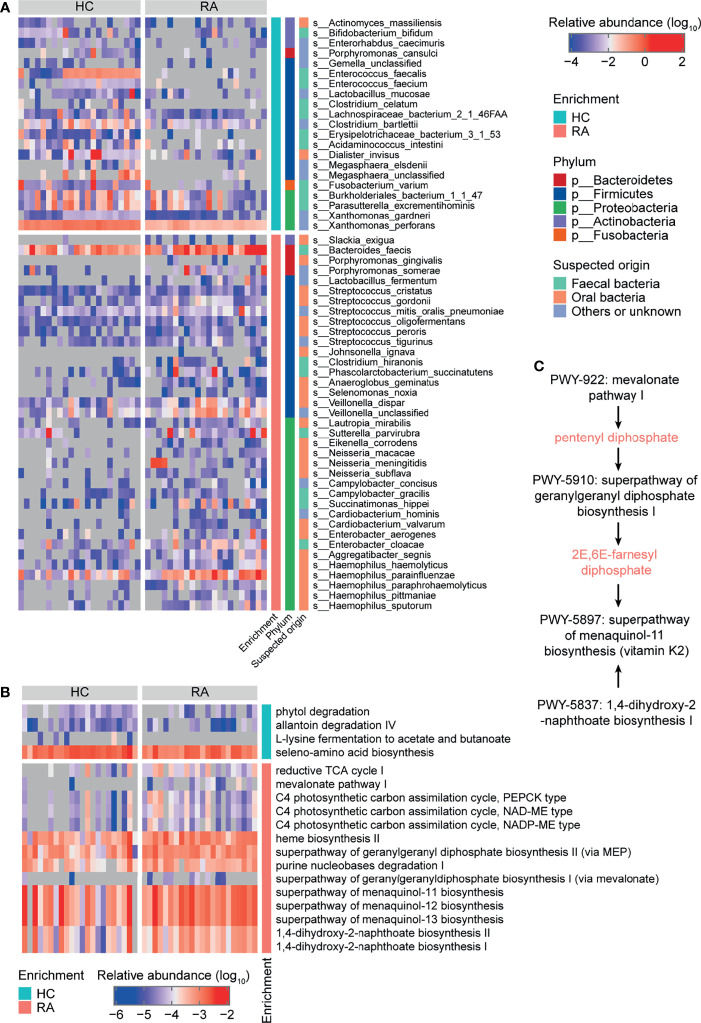
Taxonomy and function of gut microbes that varied between rheumatoid arthritis (RA) patients and healthy controls (HCs) at baseline. Heatmaps of differentially abundant **(A)** bacterial species and **(B)** MetaCyc metabolic pathways in the RA group compared to the HC group. **(C)** MetaCyc vitamin K biosynthesis-related pathways.

At baseline, 12 phyla, 225 genera, and 656 species were identified. The dominant phyla were Bacteroidetes (69.92% ± 13.95%), Firmicutes (20.88% ± 11.71%), and Proteobacteria (5.48% ± 6.47%) (**Figure S1D** and **Table S10**), and the dominant genera were *Bacteroides* (46.41% ± 22.23%), *Prevotella* (12.18% ± 22.40%), *Alistipes* (6.19% ± 6.70%), *Faecalibacterium* (3.79% ± 3.84%), and *Eubacterium* (3.06% ± 2.82%) (**Figure S1E** and **Table S11**). The number of different taxa at each taxonomic level was 1 phylum, 2 classes, 7 orders, 12 families, 26 genera, and 58 species between the HC and RA groups (based on the Wilcoxon rank-sum test). Among them, for 26 significant differences genera, 16 genera were enriched in RA patients, e.g. *Neisseria, Haemophilus, Veillonella, Campylobacter*, and 10 genera were enriched in HC, e.g. *Xanthomonas, Enterococcus, Megasphaera* (**Figure S2** and **Table S3**). The 58 species with significantly different were all low abundance species (mean relative abundance <2%) (**Figure 1A** and **Table S3**). 37 species were enriched in RA patients, including *Streptococcus* spp., *Haemophilus* spp., and *Neisseria* spp., while the remaining 21 species were depleted, including *Clostridium celatum, Enterococcus faecalis*, and *Fusobacterium varium*. Most of the RA-enriched species belonged to *Proteobacteria* (19, 51.35%), followed by *Firmicutes* (14, 37.84%). Most of the HC-enriched species belonged to *Firmicutes* (12, 57.14%). However, for *Prevotella* spp., no significant differences were observed between the HC and RA (**Figure S3**). Regarding the origin of the species, 63.16% of the RA-enriched species were residents of the human oral cavity such as the genera *Streptococcus*, *Neisseria*, and *Haemophilus*, while 47.62% of the HC-enriched species were residents of the human gut.

Regarding gut microbial function, there were 18 significantly different MetaCyc metabolic pathways in the RA patients compared to HCs (**Figure 1B** and **Table S4**). Downregulated pathways included allantoin degradation IV (anaerobic) (PWY0-41), L-lysine fermentation to acetate and butanoate (P163-PWY), and seleno-amino acid biosynthesis (PWY-6936). Upregulated pathways included purine nucleobases degradation I (anaerobic) (P164-PWY), mevalonate pathway I (PWY-922), geranylgeranyl diphosphate biosynthesis (PWY-5121 and PWY-5910), menaquinol biosynthesis (PWY-5897, PWY-5898, and PWY-5899), and 1,4-dihydroxy-2-naphthoate biosynthesis (PWY-5791 and PWY-5837) (**Figure 1B** and **Table S4**). The PWY-922 pathway is responsible for the synthesis of pentenyl diphosphate, which is then metabolized to 2E,6E-farnesyl diphosphate *via* the PWY-5910 pathway. 2E,6E-farnesyl diphosphate, along with the products of the PWY-5837 pathway, are raw materials for vitamin K2 biosynthesis. Thus, the PWY-922, PWY-5910, and PWY-5837 pathways, which are involved in vitamin K2 biosynthesis in gut microbes, were upregulated in RA patients (**Figure 1C**). These results further indicate that the gut microbial composition and function differed between RA patients and HCs.

### Associations of Gut Microbiota With Clinical Indices

Based on the above analysis, changes in gut microbes and differences in gut microbiome functional pathways between RA patients and the HCs were observed. To investigate the relationships among the clinical indices, the gut microbiome species and the gut microbiome KEGG functional pathways at baseline in the RA and HC groups, correlation coefficients were calculated using the method reported by Pedersenet et al. (23).

**Figure 2:**
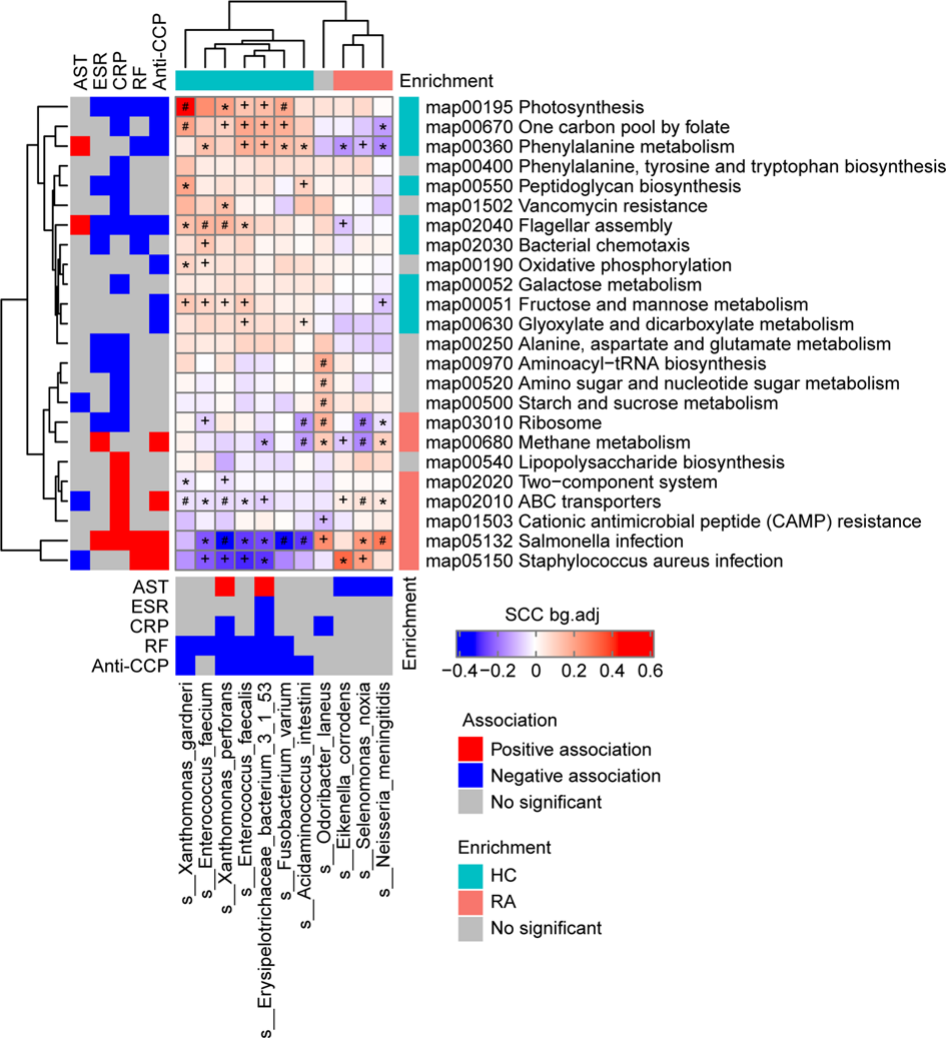
Associations among the phenotypes (rheumatoid arthritis RA-related clinical indices), gut microbiome species, and gut microbiome functions (KEGG pathways) in the RA patients and healthy controls (HCs) at baseline. Left and bottom panels show significant correlations between the RA-related clinical indices and KEGG pathways or bacterial species, respectively (blue, negative association; red, positive association; grey, no significant association). Top and right panels show the treatment group-specific enrichment of bacterial species and KEGG pathways, respectively (blue, HC-enriched; red, RA-enriched; grey, no significant difference). Middle panel shows the significant associations between the clinical index-related bacterial species and clinical index-related KEGG pathways (blue, negative; red, positive). +, FDR < 0.05; *, FDR < 0.01; #, FDR < 0.001, according to the Wilcoxon rank-sum test. AST, aspartate transaminase; ESR, erythrocyte sedimentation rate; CRP, high-sensitivity C-reactive protein; RF, rheumatoid factor;Anti-CCP, anti-cyclic citrullinated peptides.

There were 11 bacterial species and 24 KEGG pathways associated with the clinical indices (**Figure 2** and **Table S7**). 9 HC-enriched pathways and 7 HC-enriched species were negatively correlated with ESR, CRP, RF, or anti-CCP, while 7 RA-enriched pathways were positively correlated with at least one of these clinical indices. In contrast, these species and pathways exhibited the opposite correlations or no correlation with AST. In detail, the phenylalanine metabolism pathway (map00360) was identified as being positively correlated with AST and negatively correlated with anti-CCP and RF, and it was positively correlated with five HC-enriched species (*Enterococcus faecium*, *E. faecalis, Acidaminococcus intestini*, *Erysipelotrichaceae bacterium* 3-1-53, and *F. varium*) and negatively correlated with three RA-enriched species (*Eikenella corrodens*, *S. noxia*, and *Neisseria meningitidis*). Of note, *E. bacterium* 3-1-53 was positively correlated with AST, while *E. corrodens*, *S. noxia*, and *N. meningitidis* were negatively correlated with AST, and *E. bacterium* 3-1-53, *E. faecalis*, and *F. varium* were negatively correlated with RF and anti-CCP. Thus, gut microbes may affect phenylalanine metabolism and thereby influence AST and RF/anti-CCP (via mechanisms that act in opposite directions for the former *vs* the latter two). In addition, the *Salmonella* infection pathway (map05132) was identified as being positively correlated with ESR, CRP, RF, and anti-CCP, and it was negatively correlated with six HC-enriched species (*E. faecium*, *Xanthomonas perforans*, *E. faecalis, E. bacterium* 3-1-53, *Acidaminococcus intestinii*, and *F. varium*) and positively correlated with two RA-enriched species (*S. noxia* and *N. meningitidis*). Of note, *X. perforans, E. faecalis, E. bacterium 3-1-53*, and *F. varium* were negatively correlated with RF and anti-CCP, and the mechanism may involve the *Salmonella* infection pathway. These results suggest that gut microbes may influence clinical indices in the host.

### Different Effects of the Two Treatments on Clinical Indicators

**Figure 3:**
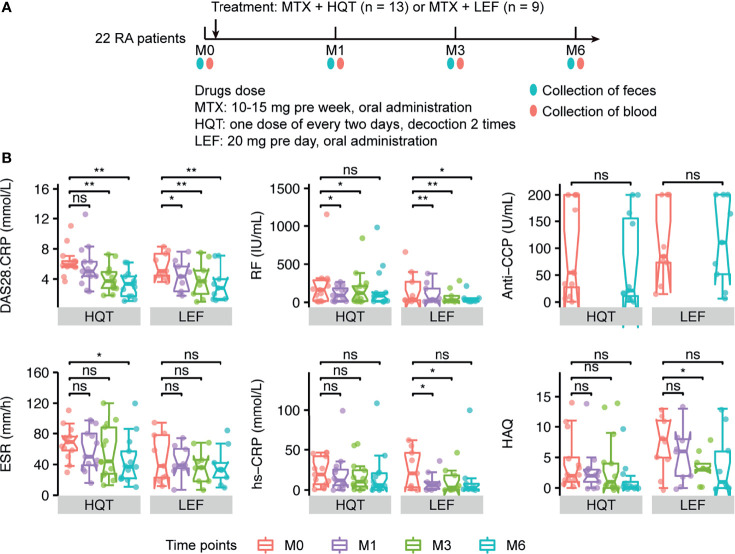
Different effects of the two treatments on primary clinical indicators **(A)** Sample collection time points in the two treatment groups. **(B)** Changes between time points in RA-related primary clinical indices in HQT and LEF groups. *P < 0.05; **P < 0.01, ns, no significance, between different time points according to Wilcoxon rank-sum test. DAS28-CRP, disease activity score for 28 joints based on the C-reactive protein level; RF, rheumatoid factor; Anti-CCP, anti-cyclic citrullinated peptides; CRP, high-sensitivity C-reactive protein; ESR, erythrocyte sedimentation rate; HAQ, health assessment questionnaires.

Furtherly, to assess the effects of different therapies on the gut microbiome in RA patients, the 13 RA patients in the HQT group received traditional Chinese medicine plus MTX and the 9 patients in the LEF group received LEF plus MTX. The RA patients underwent clinical assessment (including arthritis severity scores and inflammation-related indices) and blood and fecal sample collection at baseline and three times during the 6-month treatment period (**Figure 3A**).

Among the 22 RA patients who were treated for 6 months, most of the clinical indices were continuously and effectively improved over time compared to baseline in both the HQT and LEF groups (**Figures 3B**, **S4** and **Table S1**), including disease activity score for 28 joints based on the C-reactive protein level (DAS28-CRP), morning stiffness duration (MS), joint tenderness score (JTS), joint swelling score (JSS), RPJ, visual analogue scale for disability (VAS-D), and visual analogue scale for pain (VAS-P).

However, the two treatments influenced the clinical indices in different ways. In the HQT group, JSS, JTS, and MS were significantly reduced at month 3 compared to baseline, while these clinical indices were only significantly reduced in the LEF group at month 6. Thus, these clinical indices were improved earlier in the HQT group than in the LEF group. Additionally, in the LEF group, RF, DAS28-CRP, and RPJ were decreased at month 1 until the end of the study while, in the HQT group, RF was significantly decreased at month 1, though not at month 6, and DAS28-CRP and RPJ were significantly decreased at months 3 and 6. Moreover, ESR did not significantly change with LEF treatment, but significantly decreased at month 6 in the HQT group. In contrast, CRP was altered by LEF, but HQT had no effect. Furthermore, in the HQT group, VAS-P was decreased at month 1 until the end of the study while, in the LEF group, it only significantly decreased at month 3 until the end of the study. Health Assessment Questionnaires (HAQ) scores were not significantly changed in the HQT group, but significantly decreased at month 3 in the LEF group. Neither treatment affected anti-CCP levels (**Figures 3B**, **S4** and **Table S1**). Although many clinical indices significantly differed at various time points compared to baseline in the same treatment group, there were no significant differences between the two treatment groups. In summary, the response trends of RA-related clinical indices in the two treatment groups differed, but the clinical efficacy of the two treatments was similar.

### Effects of Treatment on the Microbiome

**Figure 4:**
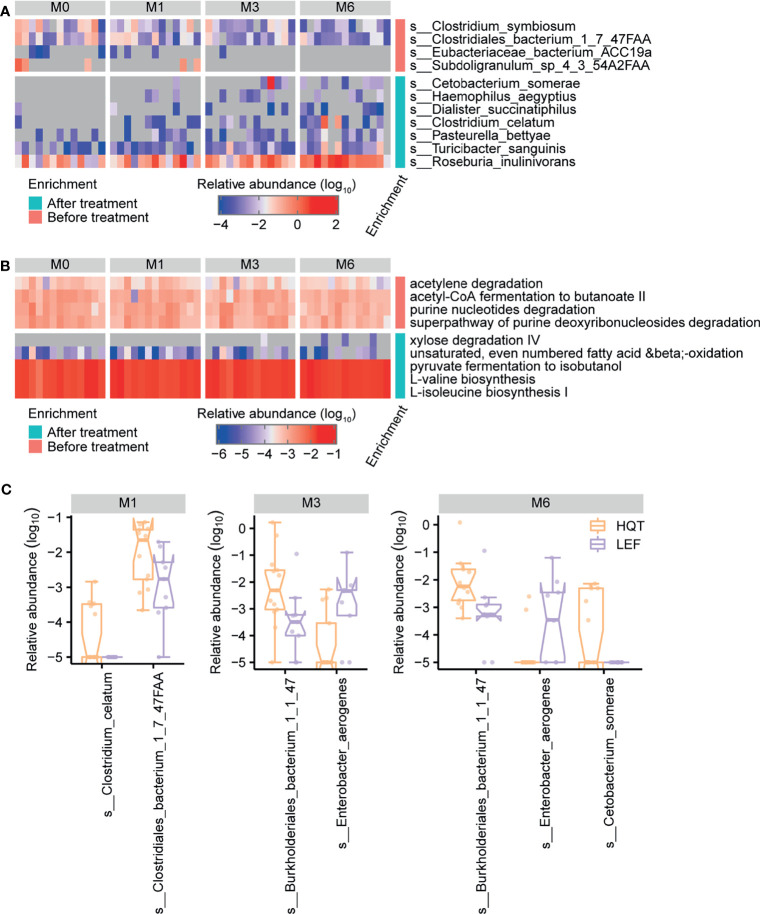
Trends over time in gut microbial taxonomy and function after treatment with Huayu-Qiangshen-Tongbi (HQT) or leflunomide (LEF). Significant changes in **(A)** species and **(B)** MetaCyc metabolic pathways in HQT group over time. P < 0.05 according to Jonckheere–Terpstra test. **(C)** Comparisons of relative abundance of various species at various time points between the HQT and LEF groups. P < 0.05 according to Wilcoxon rank-sum test.

Based on longitudinally tracking the gut microbiota during treatment, the gut microbial diversity (alpha or beta) did not significantly differ over time, except for beta diversity being greater between baseline and month 6 than between baseline and month 1 in the LEF group (**Figures S5A, B** and **Table S12**). In the HQT group, 11 species and 9 MetaCyc metabolic pathways significantly changed over time (based on the Jonckheere–Terpstra test), with 4 species (*C. somerae*, *Haemophilus aegyptius*, *Dialister succinatiphilus*, and *C. celatum*) being restored by the late stage of HQT treatment (**Figure 4A** and **Table S5**). Additionally, as HQT treatment progressed, the abundances of *Roseburiaw inulinivorans*, *Turicibacter sanguinis*, and *Pasteurella bettyae* significantly increased while *Clostridium symbiosum* and *Clostridiales bacterium* 1-7-47FAA significantly decreased (**Figure 4A** and **Table S5**). In the HQT group, bacterial purine degradation (PWY0-1297 and PWY-6353) was decreased and amino acid biosynthesis (VALSYN-PWY and ILEUSYN-PWY) was increased over time (**Figure 4B** and **Table S6**). In the LEF group, only 4 species and 2 MetaCyc metabolic pathways significantly changed over time (based on the Jonckheere–Terpstra test), but their abundances were very low in both groups (**Figure S6**, **Tables S13** and**S14**). However, the *Prevotella* spp. known as the dominant gut microbiota in RA patients was not significantly different in both the HQT-treated and LEF-treated groups. Thus, there were few changes over time in the relative abundance of gut microbiota species in the LEF group, but many changes in the HQT group.

To determine how distinct the gut microbiota in the LEF and HQT groups were, the relative abundance of each species between the LEF and HQT groups at each time point was assessed using the Wilcoxon rank-sum test. In the HQT group compared to the LEF group, *C. bacterium* 1-7-47FAA was increased at month 1, *Burkholderiales bacterium* 1-1-47 was increased at months 3 and 6, and *Enterobacter aerogenes* was decreased at months 3 and 6 (**Figure 4C**).

## Discussion

In this study, gut microbiome metagenome sequencing provided rich microbial data, including data on the microbial compositions and functions, in participants with diverse clinical phenotypes (in terms of RA-related clinical indices). We reconfirmed that certain bacteria (including oral microbiome bacteria) in the gut were associated with RA. More changes over time in the gut microbiome were observed during traditional Chinese medicine (HQT) treatment than LEF treatment. This study provides a deep understanding of the gut microbiome of RA patients during LEF or HQT treatment, and it may help to develop more efficient and safe treatments for RA.

The diversity of the gut microbiome in RA patients is a matter of ongoing debate. Some studies have reported significant differences in microbiome diversity between RA patients and healthy individuals (8, 9), but others have indicated no differences (7, 24). Our results indicated that the microbial diversity did not significantly differ between RA patients and healthy individuals, which may be caused by the small sample size and/or the involvement of RA patients with different phenotypes.

The species identified as being significantly different between RA and HCs vary among studies. Kishikawa et al. and Scher et al. reported that multiple species in the genus *Prevotella* were increased in RA patients in Japanese (24) and US (25) cohorts, respectively. In a Chinese cohort, Sun et al. found that the genera *Bacteroides* and *Escherichia-Shigella* were more abundant in RA patients (8), while Chiang et al. reported a higher abundance of the genus *Akkermansia* (9). In the present study, species such as *Streptococcus* spp., *Haemophilus* spp., and *Neisseria* spp. were found to be enriched in RA patients, while *Clostridium celatum*, *Enterococcus faecalis*, and *Fusobacterium varium* were depleted. Most of the RA-enriched species belonged to *Proteobacteria* and *Firmicutes*, and the HC-enriched species belonged to *Firmicutes*. Among them, *Enterococcus faecalis*, Yamamoto et al. found that hyperimmunization with attenuated *E. faecalis* as normal gut microbiota could provide an animal model of chronic polyarthritis (26). Chandradevan et al. and Luo et al. observed *E. faecalis* in both blood cultures and synovial tissue culture from RA patients (27, 28). However, other species and phyla have not been reported in other studies. The fact that the RA-associated species differed among these studies might reflect dietary (29) and geographical (30) variation impacting the gut microbiome composition in the various study samples.

Meanwhile, in this study, we found that most of the RA-enriched bacteria are known to colonize the oral cavity, such as *P. gingivalis*, *Aggregatibacter segnis*, *Streptococcus* spp., *Haemophilus* spp., and *Neisseria* spp. Additionally, oral microbial dysbiosis in RA patients has been reported to promote increased joint inflammation, and the oral bacteria *P. gingivalis*, *A. actinomycetemcomitans*, and *Prevotella nigrescens* are associated with RA pathogenesis (31). Moreover, oral cavity and gut bacteria in RA patients exhibit covariation. Therefore, the inflammatory status in the joints in RA patients may be due to the translocation of oral bacteria to the gut, which is consistent with our findings.

Whole-genome shotgun sequencing of the metagenome provides data on microbial function, allowing identification of altered microbial functions in RA patients relative to healthy individuals. Menaquinone, a type of vitamin K, can be produced by bacteria. Several MetaCyc menaquinone biosynthesis-related pathways were enriched in RA patients, which is consistent with previous research (32). Vitamin K homologs have been shown to affect serum CRP, matrix metalloproteinase (MMP)-3, and DAS28-CRP in RA patients (33). This indicates that the gut microbiota may contribute to the initiation or development of RA by affecting vitamin K biosynthesis.

To identify the associations of the gut microbiome species and gut microbiome functional pathways with RA-related clinical indices, correlation coefficients were calculated using the baseline data from the RA and HC groups. Most of the HC-enriched pathways and species were negatively correlated with ESR, CRP, RF, and anti-CCP, while the RA-enriched pathways and species were positively correlated with these clinical indices. Further, we concluded that gut microbes may affect phenylalanine metabolism and thereby influence the liver function index AST and RA specific index RF/anti-CCP (via mechanisms that act in opposite directions for the former *vs* the latter two). This pathway was positively correlated with HC-enriched *E. bacterium* 3-1-53, and this species was positively correlated with AST and negatively correlated with ESR, CRP, RF, and anti-CCP, which suggested that this species directly influences phenylalanine metabolism and thereby affects ESR, CRP, RF, anti-CCP, and AST. Furthermore, several gut microbial species may affect the clinical indices by influencing the *Salmonella* infection pathway. Among these species, HC-enriched species were negatively correlated with ESR, CRP, RF, and/or anti-CCP and positively correlated with AST, while RA-enriched species were negatively correlated with AST. Associations between the gut microbiota in RA patients and clinical indices were observed in another study (11), but the related gut microbiome functional pathways have rarely been reported.

By assessing the effects of different treatments on RA patients, we found that both treatments, to a certain extent, alleviated RA progression, based on different clinical indices. LEF improved the CRP level, while the traditional Chinese medicine (HQT) improved the ESR level. This result indicates that their mechanisms of action differ. Previous research reported that the gut microbiota was moderately restored in RA patients after DMARD treatment (7), and similar results were found in spondyloarthritis (34) and ankylosing spondylitis (35). We observed that several bacteria were restored after both treatments but the gut microbiota exhibited greater restoration (regarding gut microbial species and functional pathways) in the HQT group than the LEF group. A possible reason for this is that the composition of the traditional Chinese medicine (HQT) is more complicated than LEF and so has broader effects.

In a previous study, the quantification of 10 compounds in HQT extracts was carried out by UPLC-PDA method (18). The 10 compounds are danshensu, 3-caffeoylquinic acid, paeoniflorin, rutin, quercetin, salvigenin, caffeic acid, rosmarinic acid, calycosin and glycyrrhizic acid, respectively. Interestingly, most of these 10 compounds in the HQT extract were shown to have significant antibacterial effects and even to directly modulate the homeostasis of the gut microbiota. For example, Liu et al. suggested that the paeoniflorin derivative can be used as an antibacterial agent to abolish the hemolytic activity of *Staphylococcus aureus* α-toxin (36). Motallebi et al. highlighted that a combination of rutin and florfenicol could act as an alternative strategy to treat bacterial infections (37). The antibacterial effects of quercetin have been demonstrated in several studies (38, 39), and quercetin has even been found to be effective in restoring the intestinal microbiota of mice after antibiotic treatment (40). Meanwhile, chlorogenic acid was found to have antibacterial activity against Foodborne Pathogen *Pseudomonas aeruginosa* and *Salmonella enteritidis* (41), and caffeic acid was found to have antibacterial activity against *Staphylococcus aureus* clinical strains (42). In addition, the antibacterial effect of *Salvia miltiorrhiza* Bunge (Danshen), a major component herb of HQT, has been widely reported and studied (43–45). Therefore, we speculate that HQT can significantly restore and improve gut microbial species and function in RA patients, possibly due to the fact that HQT contains a variety of compounds with antimicrobial effects.

During drug treatment, there were 11 species and 9 MetaCyc metabolic pathways in the HQT group while 4 species and 2 MetaCyc metabolic pathways in the LEF group changed significantly over time. These species and pathways include *C. somerae, Clostridium symbiosum, Turicibacter sanguinis, C. celatum* and bacterial purine degradation pathways (PWY0-1297 and PWY-6353) et al. In particular, the presence and abundance of an important microbe, *C. celatum*, was restored in the HQT group. Importantly, it was shown to be downregulated in RA patients relative to HC. Currently, *C. celatum* has tended not to be noted in microbiota studies of RA patients. However, for the genus *Clostridium*, Schmidt et al. found that infection with *Clostridioides (Clostridium) difficile* VPI 10463 induced intestinal inflammation and thus reduced the incidence of collagen induced arthritis (CIA) in mice (46). Moreover, we identified several other restored species and functions that are not consistent with previous studies, possibly due to treatment differences. Recent studies have also reported that MTX treatment can alter the gut microbiota of RA patients. Nayak et al. evaluated the differences in gut microbiome between responders (MTX-R) and non-responders (MTX-NR) after MTX treatment for RA, but found that MTX-R exhibited a significant decrease in *Bacteroidetes* relative to MTX-NR, without any significant difference in the other phyla (47). Similarly, the analysis by Artacho et al. found that, at the phylum level, MTX-R was significantly more abundant in OTUs from *Bacteroides* and *Prevotella* genus (*Bacteroidetes* phylum) and less abundant in OTUs from the order *Clostridiales* and the genus *Ruminococcus* (phylum *Firmicutes*) (48). However, These microbiota were not found to change in our study of drug therapy, which may be influenced by various factors such as diet and environment. Therefore, in this study, we speculate that some of the alterations in the gut microbiota of the HQT and LEF groups were also caused by MTX.

Despite our promising findings, several limitations should be considered when interpreting the results. Accumulating studies have demonstrated that gut microbial dysbiosis can occur in RA patients compared to healthy individuals, but differences in gut microbial compositions were also observed among RA patients with different clinical phenotypes, such as ACPA seropositivity and cytokine levels (TNF-α, IL-6, and IL-17A) (8, 9). Sun et al. also reported that some basic characteristics of RA patients (including age, gender and enterotypes) alter the gut microbiota (8). The effects of geographical location on the gut microbiome community structure (30) also need to be considered. Therefore, future metagenome research on RA populations should be designed to minimize the impact of these known factors that introduce bias. Given the known heterogeneity of the gut microbiome among individuals, exploring the role of the gut microbiome in the etiology and pathogenesis of RA requires larger sample sizes to ensure sufficient statistical power. Unfortunately, current RA microbiome studies (49, 50), including the present study, are limited by their small sample sizes. Therefore, larger longitudinal cohort studies, which can provide more precise results, are required to confirm the current research results to fully understand the etiology of RA.

Our results further confirm that the initiation and/or development of RA is accompanied by alterations in part of the gut microbiota and also demonstrate that the microbiome composition and functions change during treatment, including treatment with traditional Chinese medicine. *C. celatum* was depleted in RA patients relative to HCs and it was restored in RA patients by HQT treatment. Additionally, vitamin K biosynthesis may act as a newly identified bridge between the gut microbiome and RA. Further, we observed that the two treatments had similar clinical efficacy, but the response trends of RA-related clinical indices differed between treatments. Moreover, the abundances of specific gut microbiome species in RA patients were associated with various serological and clinical indices. However, validation of these potential RA-related microbial markers using large independent cohorts is required. This is also a deficiency of the current research, and we need to consider increasing the number of samples and discussing more optimal experimental design options in depth in future studies. However, the data from this exploratory phase of the study may provide a reference for further large cohort studies. Further studies on the role of the gut microbiome in RA should incorporate other omics technologies including metatranscriptomics and metabolomics and other microbiomes such as mycobiomes and viromes. This research provides useful resources for the future development of new therapeutic strategies for RA.

## Data Availability Statement 

The datasets presented in this study can be found in online repositories. The names of the repository/repositories and accession number(s) can be found below: https://db.cngb.org/cnsa/, CNP0001832.

## Ethics Statement

The studies involving human participants were reviewed and approved by the ethics committee of Guangdong Provincial Hospital of Chinese Medicine. The patients/participants provided their written informed consent to participate in this study.

## Author Contributions

RH and QH designed the study and supervised all parts of the study. MW and ZY contributed to the clinical trial and the collection of samples and clinical data. LM conducted analyses and wrote the manuscript. All authors approved the final version.

## Funding

This study was supported by National Natural Science Foundation of China (No.81774218, No.81804041), Natural Science Foundation of Guangdong Province (No. 2021A1515011593, No. 2021A1515011477), the grant from the Clinical Research Project of Guangdong Provincial Hospital of Chinese Medicine (No. YN10101906, YN2018ML08), Guangdong-Hong Kong-Macau Joint Lab on Chinese Medicine and Immune Disease Research (2020B1212030006), Guangdong Provincial Key laboratory of Chinese Medicine for Prevention and Treatment of Refractory Chronic Diseases(2018) (No. 2018B030322012), the grant from Guangzhou Basic Research Program (No.202102010256), as well as grants from Guangdong Provincial Hospital of Chinese Medicine (No. MB2019ZZ07). The study was also funded by State Key Laboratory Project of Dampness Syndrome of Chinese Medicine (No. SZ2020ZZ17), the Key Research Project of Guangzhou University of Chinese Medicine (No. XK2019021), and the Key-Area Research and Development Program of Guangdong Province (No. 2020B1111100010).

## Conflict of Interest

The authors declare that the research was conducted in the absence of any commercial or financial relationships that could be construed as a potential conflict of interest.

## Publisher’s Note

All claims expressed in this article are solely those of the authors and do not necessarily represent those of their affiliated organizations, or those of the publisher, the editors and the reviewers. Any product that may be evaluated in this article, or claim that may be made by its manufacturer, is not guaranteed or endorsed by the publisher.
