# Supplementary material for: Sustained Drug Treatment Alters the Gut Microbiota in Rheumatoid Arthritis
Source: Front Immunol. 2021 Oct 14;12:704089. doi: 10.3389/fimmu.2021.704089 (PMC8551364; doi:10.3389/fimmu.2021.704089)
Supplement: Supplementary file 2 [file DataSheet_2.docx]

Supplementary Material

# Supplementary Methods

## DNA extraction and library construction

Fresh feces were collected by participants themselves at Guangdong Hospital of Chinese Medicine and sent to the laboratory immediately, and stored at -20 °C until transport. After transport on dry ice, fecal samples were stored at -80 °C until DNA extraction. Total bacterial DNA from each fecal sample was extracted from ~200 mg of stool with the NucleoSpin® Soil kit (Macherey-Nagel, Germany) according to the manufacturer’s instructions. Qubit (Invitrogen, USA) and 1% agarose gel electrophoresis were used to analyze the quality of DNA. The detail of DNA library construction was described as follow: 1 μg genomics DNA was randomly fragmented by Covaris and the fragmented DNAs were tested by Gel-Electrophotometric then purified by kit. The fragmented DNAs were combined with End Repair Mix, incubated at 20 ℃ for 30 min then purified by kit. The repaired DNAs were combined with A-Tailing Mix, and incubated at 37 ℃ for 30 min. Illumina adaptors were ligated to the Adenylated 3’Ends DNA, and incubated at 16 ℃ for 16 h, then purified the DNA with kit. After selecting the correct insert size DNA fragments, several rounds of PCR amplification with PCR Primer Cocktail and PCR Master Mix were performed to enrich the Adapter-ligated DNA fragments, the purified the PCR products with kit. AxyPrep Mag PCR Clean-up Kit (Axygen, USA) was used to purify DNA in all steps of DNA library construction. The final DNA libraries were used to determine the average insert size using the Agilent 2100 Bioanalyzer (Agilent Technologies, USA) and quantified by ABI StepOnePlus Real-Time PCR system (Applied Biosystems, USA).

## Metagenomic sequencing

Samples were sequenced on the Illumina HiSeq X Ten platform with an insert size of 300 bp (paired end, 150 base pairs). Before further bioinformatic analysis, raw reads containing adaptor sequence, low quality (lower Q-score 20 rate more than 50%) and ambiguous bases (N base rate less than 5%) were filtered out with SOAPnuke. 1036.69 Gb of high-quality PE reads for the 110 samples were acquired with an average of 8.22 ± 0.83 Gb (mean ± s.d.) per sample. In order to remove human host DNA contamination, reads were aligned to human genome reference (hg19) by SOAPaligner (v2.22, parameters: -m 280 -x 420 -r 1 -l 32 -s 75 -c 0.9 -v 13) and the mapping reads were discarded. The average rate of host contamination was 0.47 ± 0.97% and an average of 8.18 ± 0.83 Gb (mean ± s.d.) per sample (Supplementary Table S2).

## Metagenomic microbial community and functional profiling

The profile of microbial composition for each sample was calculated using MetaPhlAn2 (v2.0), which uses ~1M unique clade-specific marker genes (including bacterial, archaeal, viral and eukaryotic) to estimate organismal relative abundance. The parameters of MetaPhlAn2 was set as ‘--nproc 10 --stat avg_g --ignore_viruses --ignore_eukaryotes --ignore_archaea’. And then, all sample profiles were merged using merge_metaphlan_tables.py.

Functional profiling for each sample was performed using HUMAnN2 (v0.11.2). In brief, HUMAnN2 rapidly identified known microbial species in samples with MetaPhlAn2, then constructed a customized pan-genomes database which all genomes have been preconstructed and functionally annotated. Sample reads were mapped to this database with Bowtie2, with the unmapped reads were translated and mapped to a protein database (UniRef90) with Diamond. Finally, all mapping reads were used to estimate gene family abundance then annotated to metabolic enzymes to reconstruct and quantify metabolic pathways (MetaCyc). HUMAnN2 was run by the default parameters. All sample profiles were merged and renormalized using humann2_join_tables and humann2_renorm_table, respectively.

Taxa, gene families, or pathways which presented in less than 10% samples were discarded in further analysis.

# Supplementary Figures


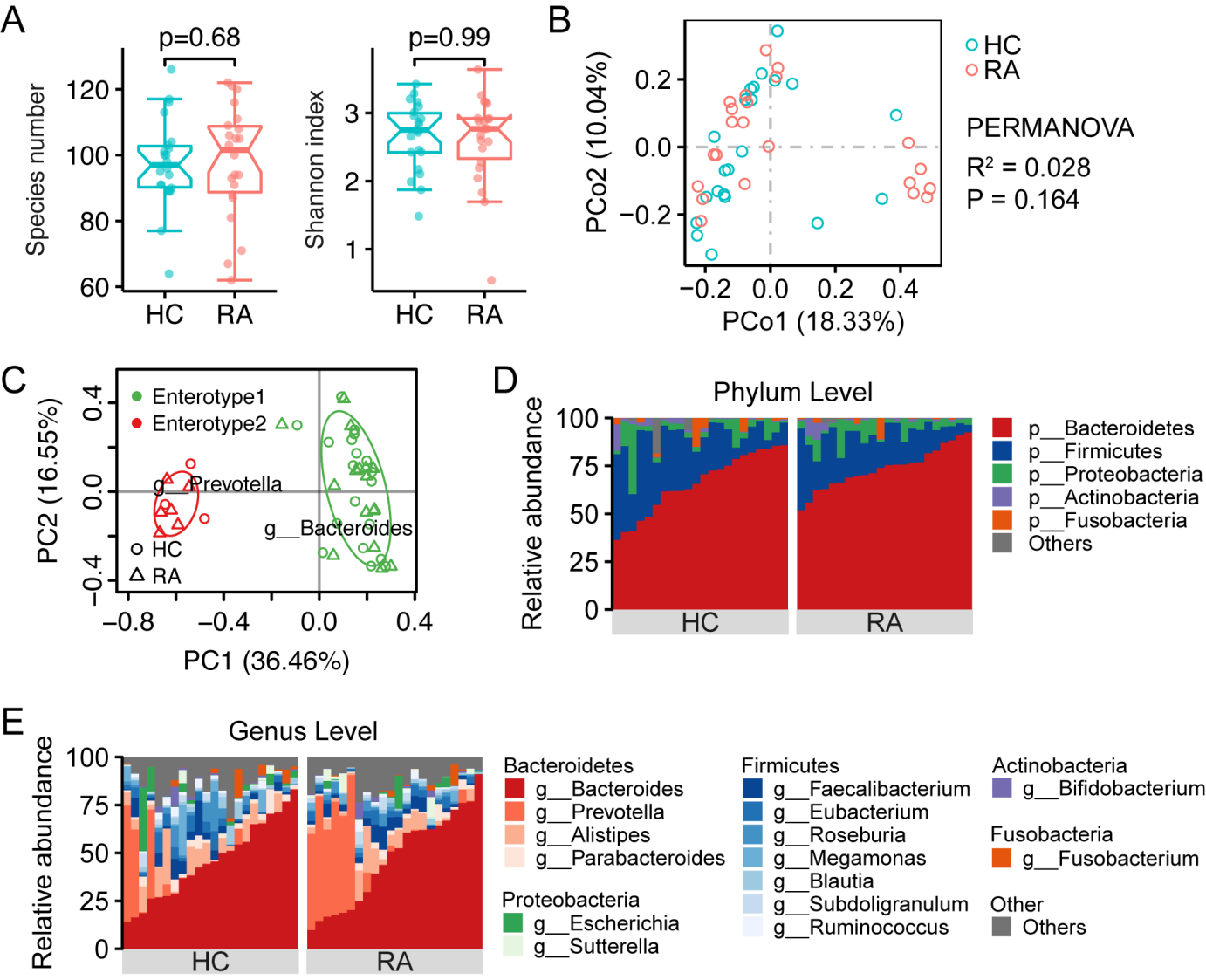


**Supplementary Figure 1.** Alpha diversity and microbial composition of healthy controls (HC) and RA patients (RA). (A) Species number and alpha diversity (Shannon index) between HC and RA samples. P value were calculated using Wilcoxon rank-sum test. (B) PCoA based on the Bray-Curtis distance on the gene family level. (C) Enterotype analysis based on the genus relative abundance profile. (D) The dominant phyla of HC and RA samples. The top 5 phyla were shown. (E) The dominant genera of HC and RA samples. The top 15 genera were shown.


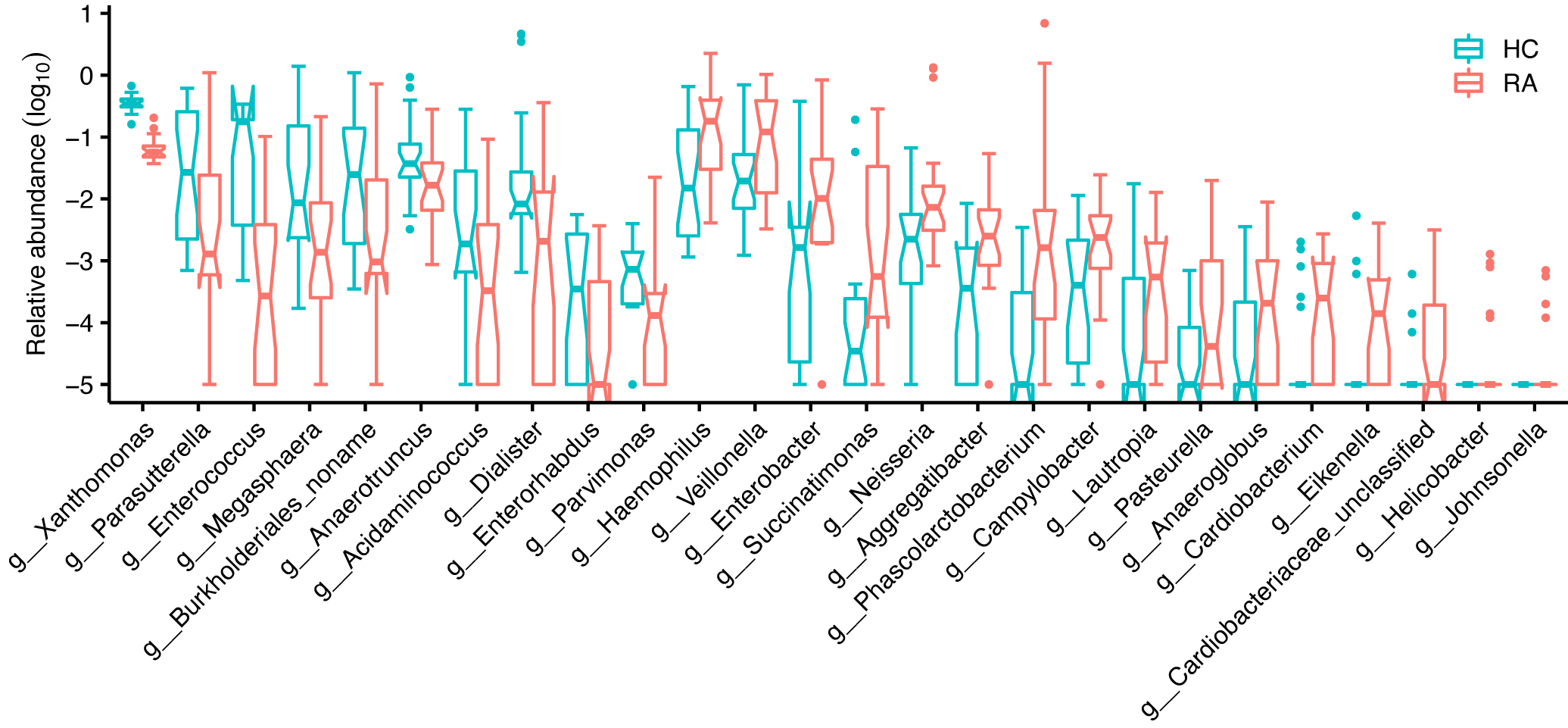


**Supplementary Figure 2.** Significant differences genera between HC and RA (Wilcoxon rank-sum test, P < 0.05).


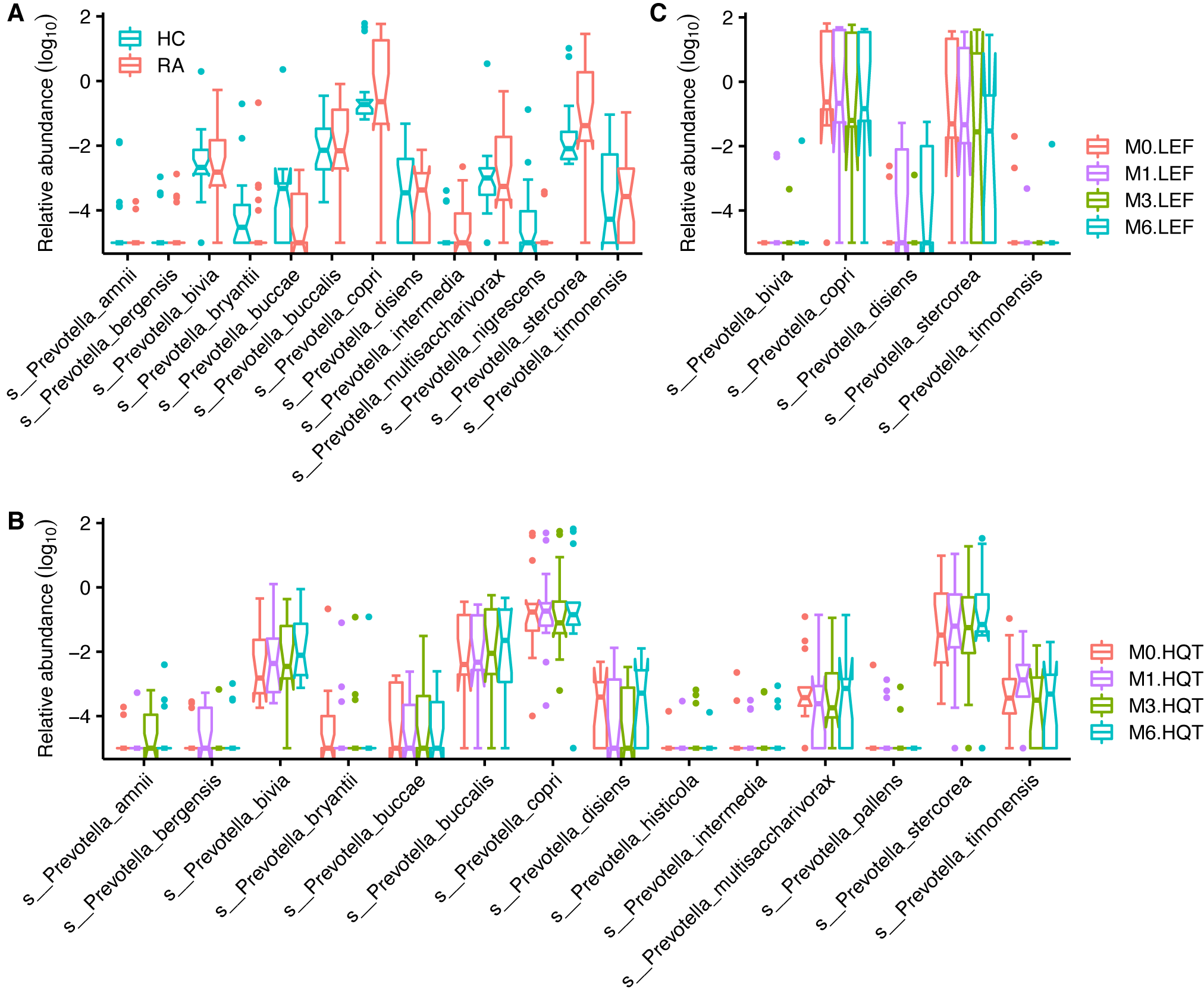


**Supplementary Figure 3.** *Prevotella spp.* relative abundance in HC-RA (A) cohort and treatment cohort (B, HQT treatment; C, LEF treatment). *Prevotella spp.* that occurrence rate in all groups less than 20% were excluded.


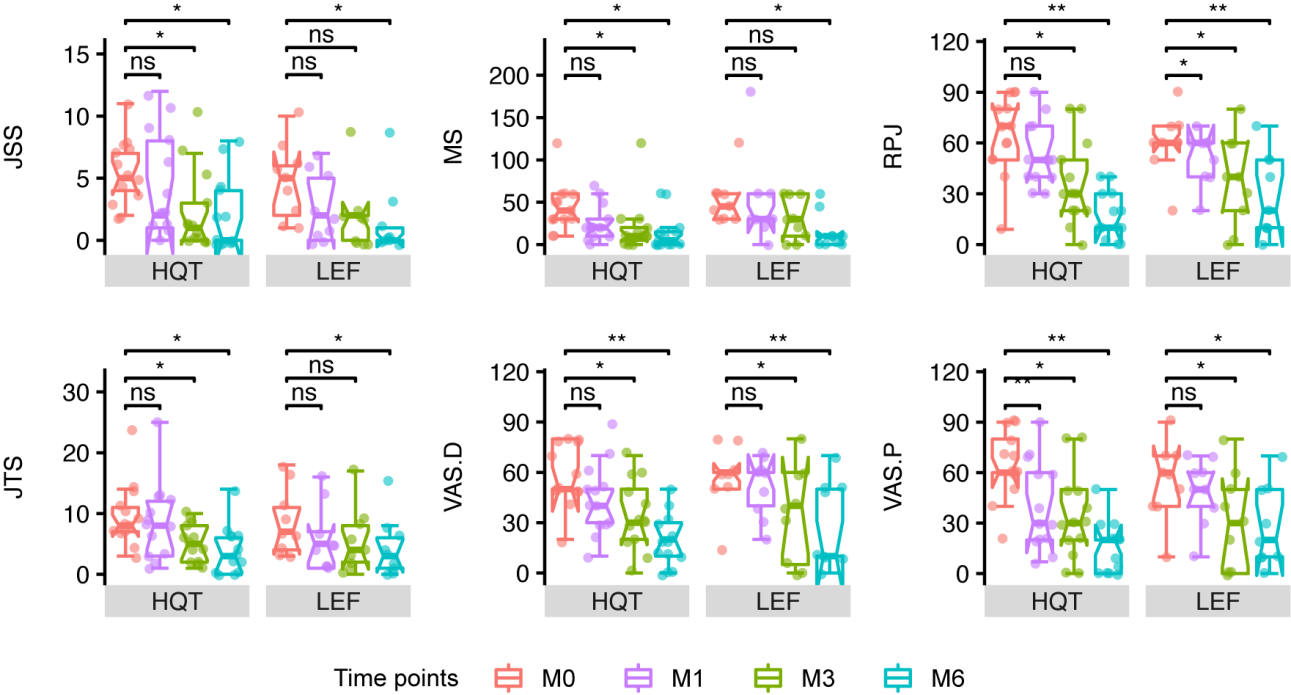


**Supplementary Figure 4.** Different effects of the two treatments on secondary clinical indicators. Changes between time points in RA-related secondary clinical indices in HQT and LEF groups. * P < 0.05; ** P < 0.01, ns, no significance, between different time points according to Wilcoxon rank-sum test. JSS, joint swelling score; MS, morning stiffness duration; RPJ, resting pain in joints; JTS, joint tenderness score; VAS-D, visual analogue scale for disability; VAS-P, visual analogue scale for pain.


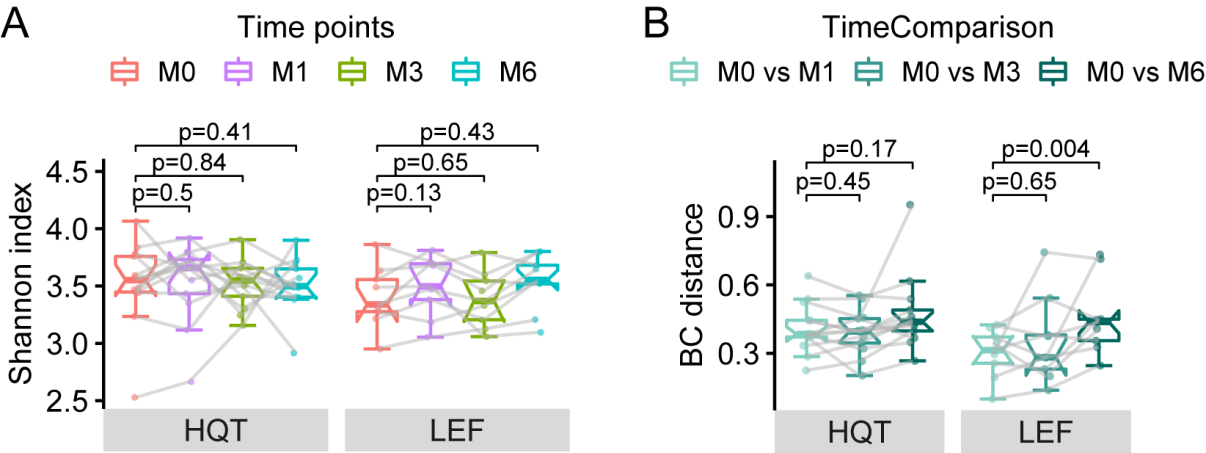


**Supplementary Figure 5.** Alteration of gut microbial composition after treatment by HQT and LEF. (A) Alpha diversity (Shannon index) between baseline and different treatment time points in RA patients treated with two drugs. P value were calculated using Wilcoxon rank-sum test. (B) Beta diversity between baseline and different treatment time points in RA patients treated with two drugs. P value were calculated using Wilcoxon singled-rank test.


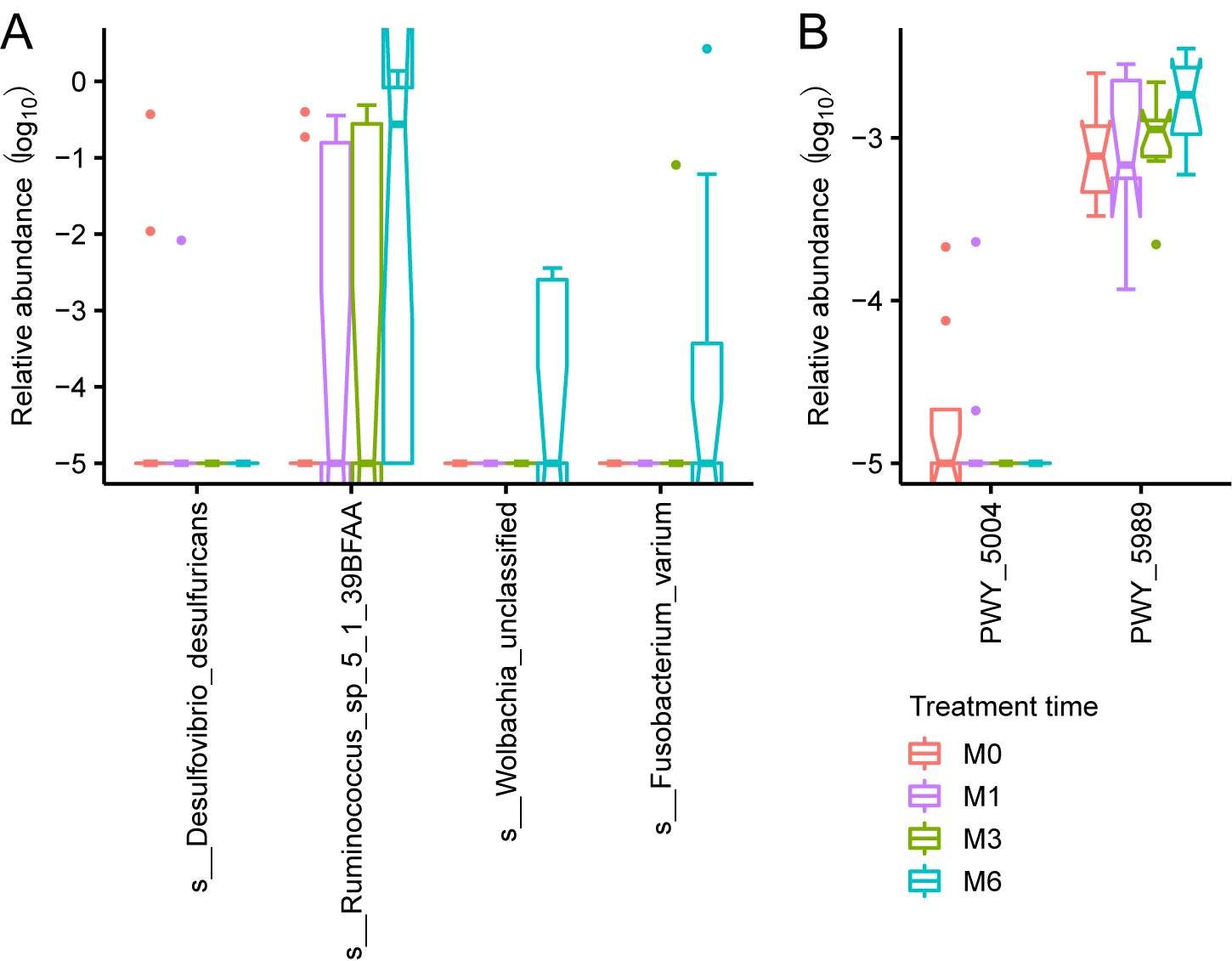


**Supplementary Figure 6.** The significant altar of species (A) and MetaCyc metabolic pathways (B) with LEF treatment progression (Jonckheere–Terpstra test, P < 0.05).
